# Photosynthetic Variability of Oblačinska Sour Cherry Ecotypes under Drought

**DOI:** 10.3390/plants11131764

**Published:** 2022-07-01

**Authors:** Marija Viljevac Vuletić, Daniela Horvat, Ines Mihaljević, Krunoslav Dugalić, Domagoj Šimić, Tihomir Čupić, Vlatka Jurković, Hrvoje Lepeduš

**Affiliations:** 1 Agricultural Institute Osijek, Južno predgrađe 17, 31000 Osijek, Croatia; ines.mihaljevic@poljinos.hr (I.M.); krunoslav.dugalic@poljinos.hr (K.D.); domagoj.simic@poljinos.hr (D.Š.); tihomir.cupic@poljinos.hr (T.Č.); 2 Croatian Agency for Agriculture and Food, Vinkovačka cesta 63c, 31000 Osijek, Croatia; vlatka.jurkovic@hapih.hr; 3 Faculty of Humanities and Social Sciences, J. J. Strossmayer University of Osijek, Ul. Lorenza Jagera 9, 31000 Osijek, Croatia; hlepedus@ffos.hr; 4 Faculty of Dental Medicine and Health, J. J. Strossmayer University of Osijek, Crkvena 21, 31000 Osijek, Croatia

**Keywords:** chlorophyll, tolerance, JIP test, relative water content, traditional cultivar, variable fluorescence

## Abstract

The selection of drought-tolerant sour cherry genotypes is essential for developing sustainable fruit production in today’s climate-change conditions. The phenotypic heterogenic population of sour cherry Oblačinska, with high and regular yield suitable for mechanical harvesting and industrial processing, is a traditional and predominant cultivar in northern Croatia (Pannonian region) and Serbia commercial orchards. In this context, 2-year old virus-free sour cherry plants of 4 isolated Oblačinska sour cherry ecotypes (OS, 18, D6, and BOR) produced by micropropagation were exposed to severe drought in a greenhouse under semi-controlled conditions to evaluate its photosynthetic intra-varietal variability. Relative water content (RWC), chlorophyll fluorescence (ChlF), and photosynthetic pigments were evaluated during the ten days of the experiment. As a visible symptom of stress, the withering of plants was followed by a diminution of RWC and photosynthetic pigments in the drought exposed leaves of sour cherry ecotypes compared to the control treatment. ChlF elucidated variability in the photosynthetic efficiency within studied sour cherry ecotypes, highlighting PI_ABS_, PI_total_, and ψE_0_ as the most sensitive and thus the most informative JIP parameters for drought screening. Among the investigated ecotypes, BOR proved to be the most sensitive. The Oblačinska sour cherry ecotype OS showed the highest tolerance to drought conditions and, therefore, can be used as a source of tolerance in sour cherry breeding programs.

## 1. Introduction

Drought is a meteorological term for a period without significant rainfall. Tolerance to stress caused by drought is present in almost all plants, but the level of tolerance varies among species and even among cultivars of the same species [1,2,3]. Depending on the intensity and duration of stressful conditions, plant species react differently at different organizational levels and developmental stages [4]. The balance of water content in the plant, i.e., cell, is determined by the loss of water through transpiration and absorption of water from the soil. Reduced water content, leaf water potential reduction, loss of turgor, and stomata closing led to photosynthesis arrest, metabolic disorders, and plant death [1]. Droughts reduced the morphological and physiological traits, the leaf water potential, and stem sap movement due to the alternation of xylem anatomical features in the apple trees [5]. Understanding the impact of drought on the association of leaf water with photosynthetic parameters is important for understanding the physiological mechanisms of drought tolerance and identifying tolerant genotypes of a species [6]. A widely used indicator of water status in plants under drought is leaf-relative water content (RWC) [7,8]. Water relations between soil and leaves correlate through the soil water content and RWC [9], suggesting drought conditions when they are reduced compared to optimal conditions.

Photosynthetic organisms change their photosynthetic activity to adapt to stressful conditions such as drought [10]. When protein-chlorophyll complexes of thylakoid membranes are damaged by stress or natural physiological changes (ripening, senescence, etc.), fluorescence as a natural process of chlorophyll molecules is altered. Chlorophyll fluorescence (ChlF) can be easily measured and analyzed by different technics. One of the widely used technics is the JIP test, which provides parameters based on the theory of energy fluxes in thylakoid membranes [11]. JIP parameters describe photosynthetic reactions through algebraic equations and quantify the characteristics of photosystems’ structure and functions. JIP parameters detect changes in photosynthetic efficiency at the cellular level even before the appearance of visible symptoms of stress [12]. The studies based on ChlF analysis on leaves have proven the impact of drought on plant photosynthetic efficiency in the passion fruit [13], the Amur Grape [14], apple [3,15], and sweet cherry [16], etc. Under drought stress, photosynthesis is limited by damage at the chloroplast level as the concentrations of chloroplast pigments, especially chlorophyll *a* and chlorophyll *b*, significantly decline [17,18,19,20,21].

Oblačinska sour cherry is a cultivar with a high and regular yield suitable for mechanical harvesting and industrial processing. It is an autochthonous, i.e., traditional, cultivar distributed in a wider area of ex-Yugoslavia, mainly in northern Croatia (Pannonian region) and Serbia. Because of its wide and long cultivation under different agro-ecological conditions and propagation, both by suckers and by seeds, it became a mixed population of different genotypes/ecotypes. Variability of Oblačinska sour cherry ecotypes is already proven on morphological, pomological, and nutritional levels [22,23,24,25,26]. At the same time, genetic analyses revealed genetic similarity at the molecular level based on SSR and AFLP markers [27]. Further, fruits of Oblačinska sour cherry ecotypes in different ecological conditions reveal significant changes in their nutritional values [28,29], indicating that adverse ecological conditions, such as temperature or rainfall, could differentially affect metabolic processes in Oblačinska sour cherry ecotypes. Given the ubiquity of climate change, understanding the morpho-anatomical and physiological changes in tolerance to drought stress can be used to select available genotypes or as a breeding tool for the creation of new genotypes which will be tolerant to drought [7,8,30,31].

Vuković [26] investigated Oblačinska sour cherry ecotypes in the comparative experiment and found that ecotype OS revealed higher yield and yield efficiency than ecotypes 18 and D6. Furthermore, the ecotype OS of the Oblačinska sour cherry cultivar was already found to be more tolerant to drought than the sour cherry cultivar Kelleris 16 [27]. The specific objective of this study was to evaluate four Oblačinska sour cherry ecotypes against drought based on visual symptoms, RWC, photosynthetic pigments concentration, and JIP test parameters to elucidate its tolerance to drought conditions. Combining these measurements allows for studying the physiological mechanisms of plant photosynthetic adaptation to drought. We hypothesized that an ecotype OS has better drought tolerance than other Oblačinska sour cherry ecotypes because of its superior morphological, pomological, and nutritive traits mentioned earlier. To the best of our knowledge, studies about the influence of drought on the photosynthesis level within ecotypes are rare. For the Oblačinska sour cherry populations, they have never been made. Furthermore, results about drought tolerance of Oblačinska sour cherry ecotypes will inform breeders and growers for future selection of plant material and the possibility of using ChlF to screen the plant material.

## 2. Results

### 2.1. Visual Symptoms of Drought Stress on Plants

Visually assessed drought-induced effect on four Oblačinska sour cherry ecotypes showed that ecotype 18 had 95% of withered plants at six days after stress (DAS), while plants of ecotype OS did not show any visual symptoms of drought (Table 1). Up to the 10 DAS, ecotypes 18, D6 and BOR plants were 100% withered, while ecotype OS had 45% of withered plants. Furthermore, although ecotype 18 had the most withered plants at 6 DAS, the first occurrence of dried plants was found at 7 DAS, and it was the most pronounced in ecotype BOR (55%). Up to the 10 DAS, in ecotypes 18, D6, and BOR, a significant percentage of the dried plants was noticed, contrary to ecotype OS.

### 2.2. Relative Water Content

**Figure 1:**
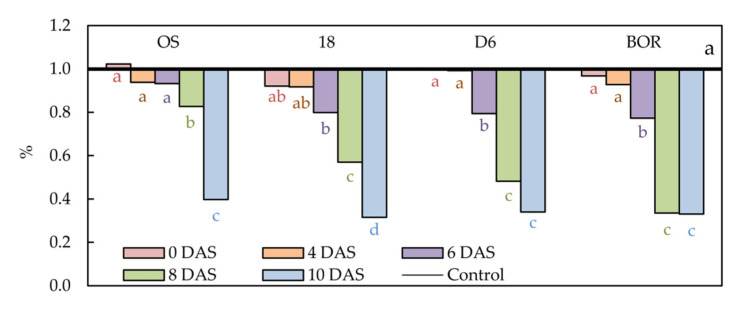
Relative water content (RWC) in the leaves of Oblačinska sour cherry ecotypes (OS, 18, D6, and BOR) under control and drought conditions was obtained at 0, 4, 6, 8, and 10 days after stress (DAS). Normalized values of drought treatments according to control treatments are presented as mean values (*n* = 5). Points labelled with different letters differ significantly at *p* < 0.05. Raw data are presented in Table S1.

A reliable indicator of the drought-induced effect on plant water status is relative water content (RWC), so its follow-up during the experiment is shown in Figure 1. The first significant decrease of RWC was found in leaves of ecotypes 18, D6, and BOR at 6 DAS (20.14, 20.62, and 22.67%, respectively) (Table S1). The decrease in RWC in these genotypes continued until 10 DAS, and it was the most significant in leaves of ecotype 18 at 10 DAS (68.50%). On the contrary, the first significant decrement of RWC in leaves of ecotype OS was found at 8 DAS (17.28%) and continued at 10 DAS to 60.22% of control values.

### 2.3. Photosynthetic Pigments

**Figure 2:**
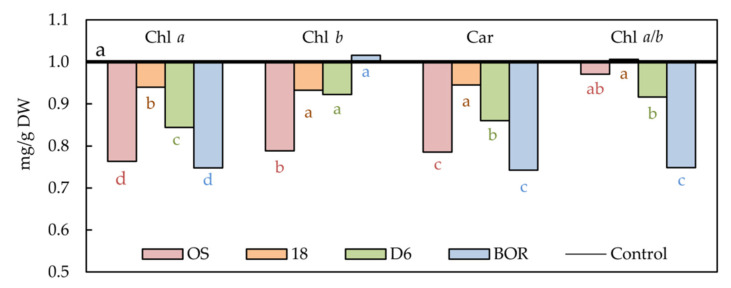
Photosynthetic pigments (Chl *a*, Chl *b*, Car, and Chl *a*/*b*) in the leaves of Oblačinska sour cherry ecotypes (OS, 18, D6, and BOR) under control and drought conditions obtained at 8 days after stress (DAS). Normalized values of drought treatments according to control treatments are presented as mean values (*n* = 5). Points labelled with different letters differ significantly at *p* < 0.05. Raw data are presented in Table S2.

The photosynthetic pigments’ concentration was strongly influenced by drought at 8 DAS compared to the control treatment (Figure 2). Chlorophyll *a* (Chl *a*) was significantly reduced by drought in leaves of all studied sour cherry ecotypes (Table S2). The most significant diminution of Chl *a* concentration was found in leaves of OS and BOR ecotypes (23.65 and 25.20%, respectively), while in ecotype 18 it was the least (6.05%). The chlorophyll *b* (Chl *b*) concentration was significantly decreased only in the leaves of ecotype OS (21.17% compared to control leaves). As for Chl *a*, carotenoids (Car) were the most decreased in leaves of OS and BOR ecotypes (21.47 and 25.74, respectively). In leaves of ecotype 18, the drought did not influence Car concentration. Drought did not impact Chl *a*/*b* in leaves of ecotypes OS and 18, while in the leaves of D6, drought decreased Chl *a*/*b* by 8.38% and BOR by 25.16% compared to control.

### 2.4. Photosynthetic Efficiency

**Figure 3:**
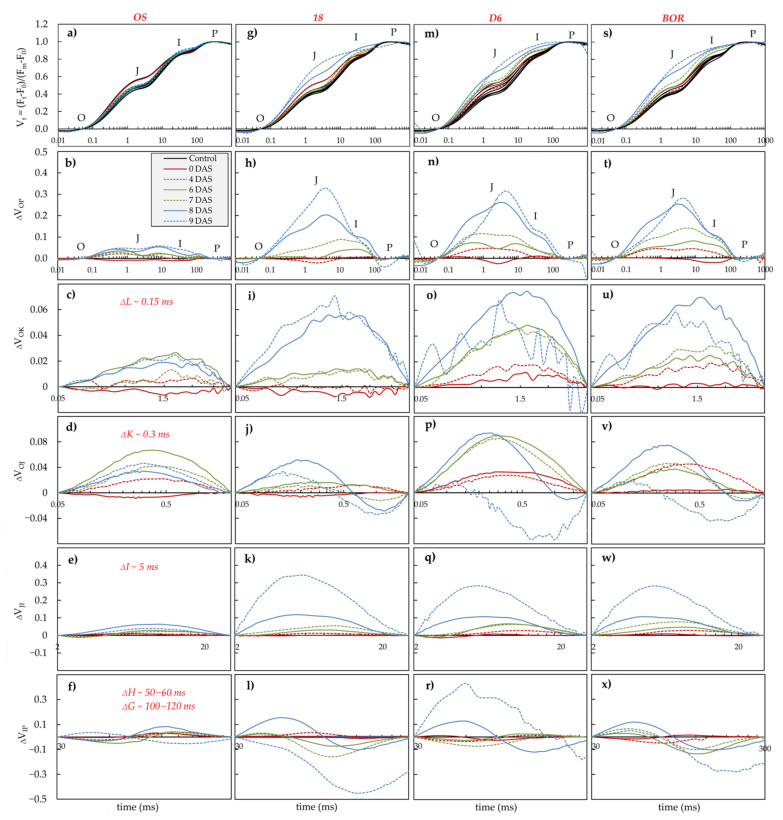
Changes in the shape of the chlorophyll *a* fluorescence transient curves in the leaves of Oblačinska sour cherry ecotypes OS (**a**–**f**), 18 (**g**–**l**), D6 (**m**–**r**), and BOR (**s**–**x**). Each curve represents the average kinetics of 20 measurements per ecotype, treatment and day after stress (DAS). The relativie variable fluorescence transient (V_t_) (**a**,**g**,**m**,**s**) shows typical O-J-I-P steps. Average fluorescence data normalized in the O–P (**b**,**h**,**n**,**t**), O–K (**c**,**i**,**o**,**u**), O–J (**d**,**j**,**p**,**v**), J–I (**e**,**k**,**q**,**w**), and J–P **(f**,**l**,**r**,**x)** phases of the OJIP transients revealed specific bands (∆L, ∆K, ∆I, ∆H, ∆G). The difference kinetics (∆V_OP_, ∆V_OK_, ∆V_OJ_, ∆V_JI_, and ∆V_JP_) in the relative variable fluorescence was calculated as ∆V_t_ = V_t_(control) – V_t_(drought) for each ecotype and each DAS.

Chlorophyll *a* fluorescence (ChlF) transients, measured at 0, 4, 6, 7, 8, and 9 DAS, in leaves of sour cherry ecotypes exposed to drought and control conditions, are presented in Figure 3. At 10 DAS, plants of ecotypes 18, D6, and BOR were 100% withered, so results of ChlF were not given because abnormal fluctuations of difference curves already occurred at 9 DAS (Figure 3j,l,o,p,u,v) as a consequence of abnormal values recorded in drought treatment, so the difference kinetic curve roamed or gave falsely negative bands. However, differences in relative variable fast fluorescence transients revealed significant variability of sour cherry ecotypes in the response of photosynthetic machinery to drought. In the drought, exposed leaves of ecotypes 18, D6, and BOR typical O-J-I-P curve shape distortion occurred at 8 DAS onward manifested as the disappearing of typical J and I peaks (Figure 3g,h,m,n,s,t).

In the drought-treated leaves of ecotype OS, a positive ΔI occurred at 6 DAS onwards and positive ΔG at 8 DAS (Figure 3e,f). Moreover, positive ΔL is visible at 6, 8, and 9 DAS (Figure 3c) and positive ΔK at 6 DAS onwards (Figure 3d). A significance of the ΔL and ΔK is demonstrated as a significant increase of V_L_ and V_K_ at 6 DAS onward (Table S3).

Drought treatment in the leaves of ecotype 18 provoked a positive ΔL and ΔK at 8 and 9 DAS (Figure 3i,j), which are proved as a significant increase in V_L_ and V_K_ at 8 and 9 DAS (Table S3). Moreover, positive ΔI occurred at 8 DAS onwards as well as positive ΔH (shifted at ~50 ms) at 8 DAS (Figure 3k,l).

A positive ΔI at 6 DAS onward in the drought exposed leaves of ecotype D6 was followed by positive ΔH (shifted at ~60 ms) at 8 DAS (Figure 3q,r), while positive ΔL is visible already at 6 DAS onward (Figure 3o) and ΔK already in 0 DAS onwards (Figure 3p). The significance of the ΔL and ΔK is demonstrated as a significant increase of V_L_ and V_K_ at 6 and 0 DAS onward, respectively (Table S3).

In the drought treated leaves of ecotype BOR, positive ΔL and ΔK are visible at 4 DAS onwards (Figure 3u,v) and proved as a significant increase of V_L_ and V_K_ at 4 DAS onward (Table S3). A positive ΔI, as well as positive ΔH (shifted at ~60 ms) occurred at 6 DAS in the drought-exposed leaves of the same ecotype (Figure 3w,x).

**Figure 4:**
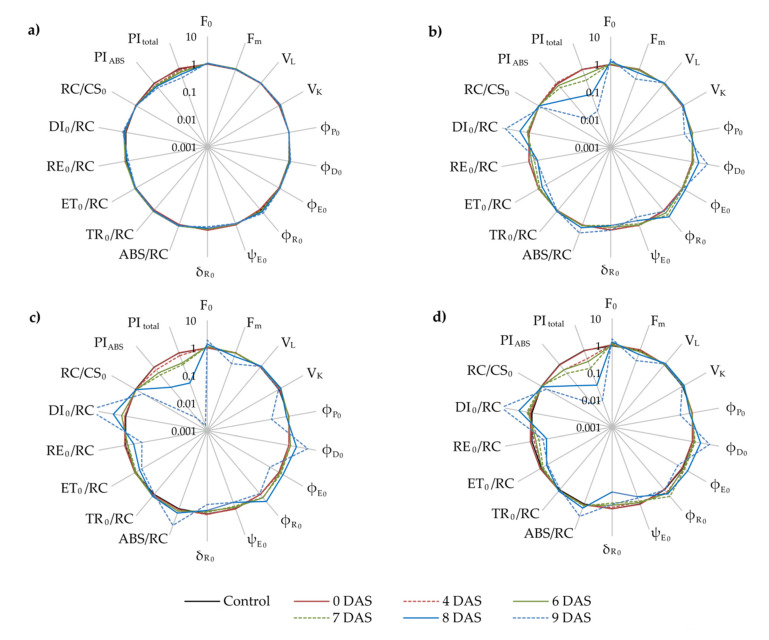
Radar plots (in logarithmic scale) of JIP parameters in the leaves of Oblačinska sour cherry ecotypes OS (**a**), 18 (**b**), D6 (**c**), and BOR (**d**) under control and drought conditions were obtained at 0–9 days after stress (DAS). Each data presents an average value of twenty measurements normalized and shown as a percentage of values in the control treatment, enabling the comparison of variables measured on different scales. Raw data shown in radar plots and statistical differences obtained by ANOVA followed by Fisher’s LSD test (*p* < 0.5) are presented in Table S3.

Changes in JIP-test parameters in drought exposed leaves of Oblačinksa sour cherry ecotypes are presented in Figure 4 as a radar plot of normalized data according to control treatment at each DAS. 

The first reaction of sour cherry leaves of ecotype OS to drought was noted at 6 DAS as an increase of minimal fluorescence (F_0_), relative variable fluorescence at 0.15 ms (V_L_), relative variable fluorescence at 0.3 ms (V_K_), absorption flux per active reaction center (RC) (ABS/RC), trapping flux per active RC (TR_0_/RC), and dissipation flux per active RC (DI_0_/RC), and decrease of density of RCs on chlorophyll a basis (RC/CS_0_) and performance index on absorption basis (PI_ABS_) (Figure 4a; Table S3). The electron transport was not affected by drought in the leaves of ecotype OS throughout the whole experiment. However, a decrease in the probability of electron transport further than QA- (ψE_0_) occurred at 9 DAS due to the TR_0_/RC increase noted at 6 DAS already. Decrease of maximum quantum yield of photosystem II (PSII) (φP_0_) and performance index for energy conservation from exciton to the reduction of photosystem I (PSI) terminal acceptors (PI_total_) at 8 DAS in leaves exposed to drought is connected with an increase quantum yield of energy dissipation (φD_0_) and quantum yield for the reduction of terminal electron acceptors at the PSI acceptor side (φR_0_) at the same day. The probability of an electron from the intersystem carriers reducing terminal electron acceptors at the PSI acceptor side (δR_0_) and electron flux reducing terminal electron acceptors at the PSI acceptor side per RC (RE_0_/RC) decreased after 9 DAS of exposure to drought suggests an adequate functioning of PSI_._

In drought-exposed leaves of ecotype 18, already at 6 DAS, alterations in the parameters related to the functioning of the PSI (an increase of φR_0_ and decrease of δR_0_ and RE_0_/RC) were noted, and they caused a decrease of PI_total_ at the same day (Figure 4b; Table S3). At 7 DAS, drought provoked an increase of φE_0_ as well as a decrease of ψE_0_, and ET_0_/RC suggested the direct influence of electron transport functioning on a decrease of the PI_ABS_ compared to control treatment. The majority of JIP parameters in drought stress leaves of ecotype 18 increased (F_0_, V_L_, V_K_, ABS/RC, TR_0_/RC, DI_0_/RC) or decreased (F_m_, φP_0_, φD_0_) compared to control leaves at 8 DAS. Only parameter RC/CS_0_ remained unchanged in drought stress leaves compared to control leaves until 9 DAS, when it decreased.

The first significant differences in drought-exposed leaves of ecotype D6 compared to control was found at 4 DAS, seen as a decrease of ψE_0_ and PI _ABS_ (Figure 4c; Table S3). F_0_, V_L_, φE_0_, φR_0_, and TR_0_/RC increased significantly at 6 DAS in drought stress treatment compared to control, while δR_0_ and PI_total_ significantly decreased on the same day. Drought significantly decreased φP_0_, ET_0_/RC, and RE_0_/RC at 7 DAS in the leaves of ecotype D6 compared to control leaves. Furthermore, at 8 DAS parameters φD_0_, ABS/RC, and DI_0_/RC increased in drought treatment compared to control treatment, while F_m_ decreased. RC/CS_0_ remained the same in drought-exposed leaves until 9 DAS compared to control leaves.

The earliest reaction to drought in leaves of ecotype BOR was noted at 4 DAS. It was seen as increased parameters V_L_, V_K_, and TR_0_/RC and decreased parameters ψE_0_, PI_ABS_, and PI_total_ in drought exposed leaves compared to control leaves (Figure 4d; Table S3). Alterations in photosystems continued at 6 DAS with a decrease of F_m_ and RE_0_/RC and an increase of φR_0._ However, at 7 DAS drought decreased (φP_0_ and ET_0_/RC) and increased (F_0_ and φD_0_) JIP parameters in drought treated leaves compared to control ones. At 8 DAS ABS/RC and DI_0_/RC increased, while at 9 DAS RC/CS_0_ decreased in drought treatment compared to control.

### 2.5. Principal Component Analysis (PCA)

**Figure 5:**
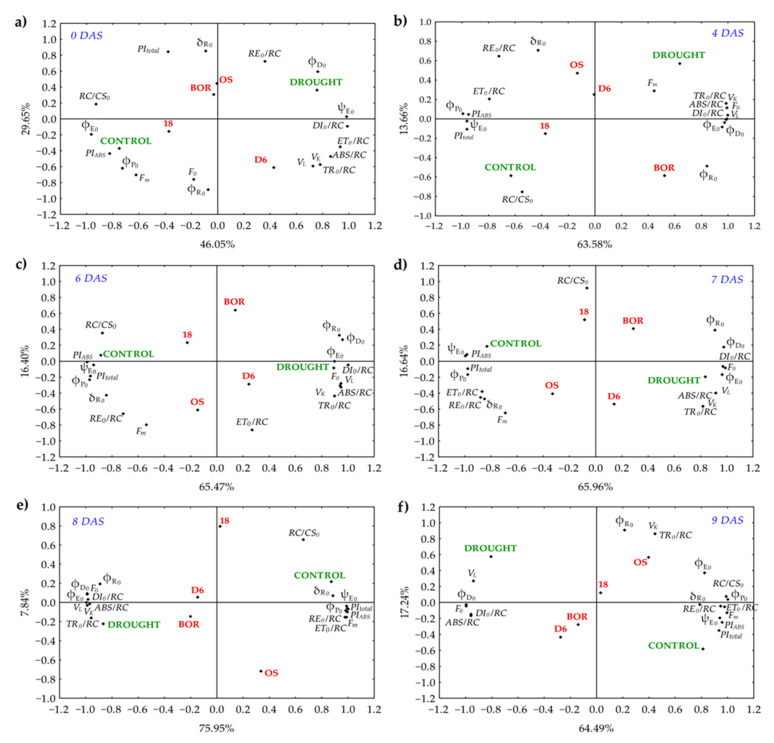
Results of principal component analysis of JIP parameters in the leaves of Oblačinska sour cherry ecotypes (OS, 18, D6, BOR) under control and drought conditions were obtained at 0 (**a**), 4 (**b**), 6 (**c**), 7 (**d**), 8 (**e**), and 9 (**f**) days after stress (DAS).

The PCA was performed on a correlation matrix of the JIP parameters for each experimental day separately (0, 4, 6, 7, 8, and 9 DAF) with the aim (1) to find parameters, which indicate the onset of drought symptoms, (2) to separate sour cherry ecotypes according to drought tolerance, and (3) to explain relations among the JIP parameters in the drought response (Figure 5). First two extracted principal components explained 75.7% of total variability at 0 DAS, 77.24% at 4 DAS, 81.87% at 6 DAS, 82.6% at 7 DAS, 83.79 at 8 DAS, and 81.73% at 9 DAS (Table S4).

At 0 DAS (Figure 5a), parameters were grouped as follows: specific energy fluxes (ABS, TR_0_, ET_0_, DI_0_, RE_0_ per RC) at the right side closely correlated with drought, and quantum yields (φP_0_, φE_0_, and φR_0_), and performance indexes at the left side of biplot closely correlated with control. Ecotypes OS, 18, and BOR were on the left side of the biplot, while D6 separated on the right side. Response of ecotypes to drought changed the arrangement of JIP parameters on the biplot during the experiment in the way that parameters PI_ABS_, PI_total_, φP_0_, F_m_, δR_0_, ψE_0_, ET_0_/RC, RE_0_/RC, and RC/CS_0_ stayed in the correlation with control conditions while φD_0_, φR_0_, F_0_, V_L_, V_K_, ABS/RC, and DI_0_/RC correlated with drought conditions (Figure 5b–f). From the 6 DAS onward (Figure 5c–f), ecotypes D6 and BOR were placed closer to drought conditions while OS and 18 were on the same side of the biplot in correlation with control conditions but distanced to opposite biplot quadrants. Drought significantly influenced parameters ABS/RC and DI_0_/RC, which strongly positively correlated during the experiment. A very high significant positive correlation was found between performance indexes (PI_ABS_ and PI_total_) and ψE_0_.

## 3. Discussion

Our study exposed plants of four Oblačinska sour cherry ecotypes to progressive drought to study their responses and adaptive mechanisms of photosynthetic apparatus to these adverse conditions. Drought provokes the accumulation of Reactive Oxygen Species (ROSs), and therefore, plants activate the antioxidative response of enzymes and secondary metabolites to protect from the abnormal conditions (i.e., stress) and to stabilize metabolism [32]. Stress effects on the plants are dependent on the duration and severity of the drought conditions [4,33]. RWC distinguished OS between other ecotypes as ecotype that retained constant RWC as in control treatment for the longest time despite unfavorable conditions indicating that it may possess higher tolerance to drought than other ecotypes. Our results corroborate the studies on pear, olive, and strawberry plants exposed to drought, where RWC discriminated cultivars according to their tolerance to leaf dehydration [7,34,35]. However, despite the most stable RWC in drought treated plants within studied ecotypes, the ecotype OS showed the most significant reduction in Chl *a* and *b* and Car as well, but without significant influence of drought on the Chl *a*/*b*. Furthermore, ecotypes D6 and BOR revealed a significant decrease of Chl *a/b,* which occurred due to a significant reduction in Chl *a.* Significant decreases of Chl *a* and *b* as a response to drought was previously found in the leaves of sweet cherry seedlings [36], pear [37] and pear rootstock seedlings [38], apple seedlings [3], *Lycium ruthenicum* Murr. seedlings [17], etc. Significant decline of the Chl *a/b* in the drought exposed leaves of ecotype BOR suggests that due to the significant reduction of Chl *a*, Chl *b* has taken a leading role as an absorption molecule in the functional antenna chloroplast of the light-harvesting complex [39]. The decline of Car in the drought-exposed leaves of sour cherry ecotypes may imply that the xanthophyll cycle was not activated properly to protect PSII from photoinhibition [40].

An earlier study on Norway spruce ecotypes identified significant differences in photosynthetic efficiency within ecotypes originating from different geographic locations (high-mountain and lowland) after one prolonged period of drought only for JIP parameters ABS/RC and DI_0_/RC [41]. Opposite that, we found that all JIP test parameters presented in our study responded to drought in the leaves of ecotypes 18, D6, and BOR. Only two parameters, φE_0_ and ET_0_/RC, in the drought exposed leaves of ecotype OS, remained unchanged during the experiment, suggesting an effective electron transport chain in PSII despite unfavorable conditions. This claim is corroborated by the fact that ψE_0_ in the leaves of ecotype OS was not reduced by drought until 9 DAS, as well as δR_0_ and RE_0_/RC, which further suggests that electron transfer to final electron acceptors at PSI was also efficient.

The first reaction of the photosynthetic machinery to drought was found in the leaves of ecotype BOR at 4 DAS, seen as positive ΔL and ΔK occurrence. This suggests impaired energetic connectivity between PSII units and impaired electron flow between the oxygen evolving complex (OEC) and the acceptor side of the RC [42]. A similar response to drought was found in the apple leaves under severe drought conditions [15] and in the leaves of modern apple and sweet cherry cultivars, suggesting higher drought tolerance of some autochthonous cultivars [3,16]. A similar pattern in OJIP curves was found in all investigated ecotypes, but it was revealed at 6 DAS in OS and D6 and 8 DAS in the leaves of ecotype 18.

The first significant impact of drought in the leaves of ecotype OS was found at 6 DAS. An increase of F_0_, with an increase of V_L_ and V_K_, and consequently, the appearance of positive ΔL and ΔK indicate impaired connectivity and system stability as well as impaired electron flow between the oxygen evolving complex (OEC) and the acceptor side of the RC [42]. However, in our study, according to earlier conclusions about electron transport in the drought exposed leaves of ecotype OS, positive but small amplitudes of ΔL and ΔK did not significantly influence electron flow because of optimal linear electron flow from PSII to PSI (δR_0_ and RE_0_/RC). The mutual interaction of significant changes in ABS/RC, TR_0_/RC, and DI_0_/RC resulted in decreasing PI_ABS_ in the drought treated leaves of ecotype OS at 6 DAS. Moreover, the fact that PI_total_ remained the same until 8 DAS can be attributed to optimal linear electron flow from PSII to PSI (δR_0_ and RE_0_/RC) and increased φR_0_. The decrease of PI_total_ and φP_0_ at 8 DAS coincide with the appearance of positive ΔG in the drought exposed leaves of ecotype OS, suggesting that, at this point, PSI was limited by blocked electron flow further than the reduced Q_A_ [43] and the redirection of linear electron flow to cyclic flow around PSI [44]. Generally, most alterations in the photosynthetic process occurred at 8 DAS in the drought-exposed leaves of ecotype OS compared to control leaves.

Unlike the OS ecotype, which maintained efficient electron transport through both PSII and PSI up to 8 DAS, the PI_ABS_ and PI_total_ reduced due to inefficient electron transfer (ET and RE) in ecotype 18 occurred earlier (7 and 6 DAS, respectively). Alterations in δR_0_, φR_0_, and RE_0_/RC at 6 DAS suggested an imbalance between the reduction and oxidation of Q_A_ [45]. The decrease of φP_0_ observed at 8 DAS, altogether with an increase of ABS/RC and DI_0_/RC, coincided with the occurrence of positive ΔK and ΔL, indicating weaker connectivity between adjacent PSIIs at the level of antenna complexes and problems with electron transfer reactions at donor and acceptor sides of PSII. The positive ΔK and ΔL also coincided with a decrease of φP_0_ after five days of exposure to drought in the leaves of modern sweet cherry cultivar compared to autochthonous cultivar [15]. In general, electron transport alterations in PSI and PSII, seen at 6 and 7 DAS, respectively, indicate that electron transport through the PSI is the most sensitive part of the photosynthetic process in the leaves of ecotype 18 exposed to drought.

In the drought-exposed leaves of ecotypes D6 and BOR, a significant decrease in PI_ABS_ was found at 4 DAS, a decrease in ψE_0_ in both ecotypes and an increase in TR_0_/RC, V_L_, and V_K_ only in the leaves of ecotype BOR. Those alterations are in accordance with the decrease of PI_ABS_ and ψE_0_ as well as the occurrence of positive ΔK and ΔL in the drought exposed leaves of sensitive sweet cherry cultivar [16]. PI_total_ in the drought exposed leaves of ecotype D6 remained unchanged until 6 DAS, when its decrease was caused by changes in the parameters describing the electron transport chain, suggesting that it is the weakest part of the photosynthetic process. Those alterations at 6 DAS coincide with positive ΔI, suggesting a reduced rate of electron transfer from Q_A_ to Q_B_ at the acceptor side of PSII and positive ΔK indicate impaired photosynthetic electron flow from PSII to PSI [46].

A diminution of PI_total_ and PI_ABS_ in the drought-exposed leaves of ecotype BOR was already found at 4 DAS. Those decreases coincide with the first changes on the OJIP curve and the occurrence of positive ΔK and ΔL. The increase of TR_0_/RC at 4 DAS in drought-exposed leaves compared to control leaves did not influence ET_0_/RC or DI_0_/RC but decreased ψE_0_. Therefore, a weak point in the leaves’ photosynthesis of the ecotype BOR under drought stress is the inability to release excess electronic excitation through usual pathways (photochemistry and fluorescence), so excitation undergoes direct losses as heat or transfers to other molecules, in this case probably carotenoids [47].

φP_0_, PI_ABS_, and PI_total_ have widely used JIP parameters in the detection of plant response to drought and therefore declared as indicators of drought [3,7,13,16,17,18,48,49]. A very high correlation of φP_0_, PI_ABS_, and PI_total_ with control treatment, immanent from 6 DAS onward, obtained by PCA analysis in our research confirmed that the specified parameters could be used as drought-stress indicators within sour cherry genotypes. According to our results, φP_0_ is a less sensitive indicator of drought stress in sour cherry seedlings than PIs, which corroborates previous research on the bean, apple, sweet cherry, etc. [3,16,48]. Besides φP_0_ and PIs, ψE_0_ was a very sensitive indicator of drought stress from the 4 DAS onward, implying that electron transport is the most sensitive part of the photosynthetic process in this type of stress.

All above mentioned explanations of investigated parameters and groupings at the same side of the PCA biplot with drought treatment suggest that ecotypes BOR and D6 were the most sensitive among sour cherry ecotypes exposed to drought. In the ecotype OS, photosynthetic efficiency was optimal for the longest time despite exposure to drought, so ecotype OS can be declared the most drought tolerant among the studied ecotypes and can be a good source of genetic tolerance in the sour cherry breeding programs.

## 4. Materials and Methods

### 4.1. Plant Material

Four ecotypes of Oblačinska sour cherry cultivar (OS, 18, D6, and BOR) were analyzed in this study. Ecotypes OS, 18, and BOR originated from Slavonia, Croatia, while ecotype D6 originated from Serbia. Morphoagronomical and pomological characteristics of investigated ecotypes are previously described [22,23,24,25,26,27,28,29]. Experiments were performed on micropropagated sour cherry seedlings produced in vitro by meristem isolation from axillary buds following the micropropagation protocol [50]. Buds were taken from the experimental orchard Tovljač, Agricultural Institute Osijek, Croatia. In brief, bud surface sterilization was made by dipping the buds in 70% ethanol for a few seconds and immersion in 1% sodium hypochlorite for 20 min. After sterilization, the meristems were isolated and transferred into the nutrient medium, supplemented with macro and microelements and plant hormones. A detailed micropropagation protocol is shown in Table S5. Development of explants in culture was carried out in an air chamber at 25 °C, photoperiod of 16 h of light (light intensity of 3000 lux) and 8 h of darkness. After a month, uninfected explants were transferred to the multiplication medium and propagated in five cycles until enough plant material was produced. Then, plantlets were transferred to the rooting medium, and after two weeks, when they had put down roots, they were planted in sterile soil and transferred to the greenhouse for acclimatization.

### 4.2. Greenhouse Experiment

The experiment was set up in a greenhouse of the Agricultural Institute Osijek. After acclimatization, plants were regularly watered, fertilized and treated against pests. Uniform two-year-old in vitro propagated sour cherry plantlets in pots (~1.2 L of Floradure substrate, Floragard, France) were selected randomly and placed on four movable tables with an automatic irrigation system in the greenhouse in a randomized complete block design with two treatments and two replicates. One replicate consisted of 10 plants of each ecotype, meaning that 20 plants per ecotype represented each treatment. According to the optimized cultivation process, plants in the control treatment were treated with the optimal amount of water every other day. Plants in drought treatment were exposed to drought stress due to a lack of water. Within ten days of the experiment, daily greenhouse temperatures varied from 20 to 35 °C, while humidity varied from around 75% in the morning to 35–40% during the day. Described conditions led to the complete drying and decay of plants exposed to water deficiency, i.e., drought stress. The percentage of wilted and dried plants within the treatment was determined by daily visual inspection of whole plants and expressed as the percent of wilted or dried plants in treatment. All samplings and measurements were conducted using completely expanded leaves (the third or fourth leaf from the apex).

### 4.3. Chlorophyll a Fluorescence Transient

Chlorophyll fluorescence (ChlF) transient was measured at 0, 4, 6, 7, 8, and 9 days after stress (DAS) on one leaf per plant on all plants in the experiment (20 measuring per treatment per ecotype). Measurements were performed at 9:00 a.m. when the temperature was 24.8 ± 2 °C, humidity 67.5 ± 10%, and 300 ± 50 μmol/m^2^s during the whole experiment. The polyphasic ChlF was induced by saturating red light (peak at 650 nm, 3000 μmol/m^2^s) on the leaves previously adapted to dark for 30 min by special leaf clips. Changes in fluorescence were recorded by the Plant Efficiency Analyzer (Handy PEA, Hansatech Instruments Ltd., King´s Lynn, UK) for 1s and fluorescence signals were collected from 10 μs up to 1 s with data acquisition every 10 μs for the first 300 μs, then continued every 100 μs up to 3 ms, and later every 1 ms, with 118 points within 1 s in total. Fast chlorophyll fluorescence rise was visualized as relative variable fluorescence (V_t_), and curves plotted as the difference in relative variable fluorescence (between O and P; O and K; O and J; J and I; and I and P) recorded in drought treated leaves from those recorded in the control treatment (∆V_t_ = V_t_(control) − V_t_(drought)) at each DAS. The characteristic peaks (bands) in the relative variable fluorescence curves appear at ~0.15 ms (∆L), ~0.3 ms (∆K), ~5 ms (∆I), ~50–60 ms (∆H), and ~100–120 (∆G) [46,51,52]. Furthermore, the obtained fluorescence data were used in the OJIP test to calculate several biophysical parameters of PSII functioning (Table S6) previously described by Strasser et al. [10,53] and Goltsev et al. [11].

### 4.4. Relative Water Content

Relative water content (RWC) in leaves was measured every two days of the experiment on five leaves per treatment per ecotype. Leaf discs (1 × 1 cm) were excised and fresh weight (FW) was immediately recorded. Leaf discs were soaked for 24 h in distilled water at 8 °C in the dark, after which the turgid weight (TW) was weighted. After drying for 24 h at 80 °C total dry weight (DW) was recorded. RWC was calculated according to the formula: RWC (%) = (FW − DW)/(TW − DW) × 100.

### 4.5. Determination of Photosynthetic Pigments Concentration

Concentrations of the photosynthetic pigments were measured at 8 DAS of the experiment. The sample (5 fresh leaves per treatment per ecotype) was powdered with liquid nitrogen in the presence of magnesium hydroxide carbonate. Photosynthetic pigments were extracted from about 0.1 g of homogenized leaf tissue with absolute ice-cold acetone for 15 min. After centrifugation (14,000× *g*, 4 °C, 10 min), the supernatant was collected, and reextractions were performed under the same conditions until the tissue became colorless. Chlorophyll *a* (Chl *a*), chlorophyll *b* (Chl *b*), and carotenoids (Car) were determined spectrophotometrically (Specord 200, Analytic Jena, Jena, Germany) at 470, 644.8, and 661.6 nm in joined supernatants and chlorophyll *a/b* ratio was calculated [54]. Concentrations of photosynthetic pigments were calculated according to the determined dry mass of samples and expressed as mg/g of DW.

### 4.6. Statistical Analysis

Statistical differences between all determined parameters in leaves of four sour cherry ecotypes at six time points (0, 4, 6, 7, 8, and 9 DAS) were analyzed using analysis of variance followed by post hoc Fisher’s Least Significant Difference (LSD) test. To minimize bias in comparisons of the ecotypes, which differed in drought tolerance, all data were normalized to control values. Data presented in the text, figures, and tables are means ± SD of twenty replicates for JIP parameters and five replicates per treatment for RWC and photosynthetic pigments concentration. Differences were considered significant at *p* < 0.05. Correlations among JIP parameters were explored by principal component analysis (PCA) to distinguish parameters that could be drought indicators. Statistical analyses and data visualization were performed in Statistica 14.0 (TIBCO Software Inc., Palo Alto, CA, USA) and Microsoft Office Excel 2010.

## 5. Conclusions

Raising plantations with drought-tolerant cultivars is important and, in the future, will be an even more important tool for farmers in food production. Therefore, the evaluation and tolerance data of the plant assortment is an important task for scientists/breeders today. Given that the production of fruit seedlings and their evaluation is a long-term process, our work has shown that in vitro produced seedlings can be used to evaluate ecotypes for their drought response. Moreover, monitoring the ChlF, i.e., photosynthetic efficiency in drought conditions, can provide detailed data on the effect of drought on a photosynthetic apparatus and, therefore, plant drought tolerance. Our research found significant photosynthetic intra-specific variability in sour cherries exposed to drought conditions. Among the investigated ecotypes, BOR proved to be the most sensitive. The Oblačinska sour cherry ecotype OS, as we hypothesized, showed the highest tolerance to drought conditions and, therefore, can be used as a source of tolerance in sour cherry breeding programs.

## Figures and Tables

**Table 1:** Percentage of withered and dried (data in brackets) drought treatment plants from the 5th to the 10th day of the experiment (*n* = 20).

Ecotype	6 DAS	7 DAS	8 DAS	9 DAS	10 DAS
OS	0	0	20	25	45
18	95	100 (10)	100 (30)	100 (60)	100 (75)
D6	60	80 (15)	90 (55)	100 (85)	100 (85)
BOR	70	85 (55)	95 (70)	95 (75)	100 (85)

## Data Availability

All data are contained within the article.

## Supplementary Materials

The following supporting information can be downloaded at: https://www.mdpi.com/article/10.3390/plants11131764/s1, Table S1: Original and normalized values of the relative water content (RWC) measured in the leaves of Oblačinska sour cherry ecotypes OS, 18, D6, and BOR under control and drought treatment obtained at 0, 4, 6, 8 and 10 days after stress (DAS). Significant differences between control and drought treatments were labelled by different letters (ANOVA LSD, *p* ≤ 0.05). Values are represented as means ± SD (*n* = 5); Table S2: Original and normalized values of the content of the photosynthetic pigment (Chl *a*, Chl *b*, Car, and Chl *a/b*) measured in the leaves of Oblačinska sour cherry ecotypes OS, 18, D6 and BOR under control and drought treatment at 8 days after stress (DAS). Significant differences between control and drought treatments were labelled by different letters (ANOVA LSD, *p* ≤ 0.05). Values are represented as means ± SD (*n* = 5).; Table S3: Original and normalized chlorophyll *a* fluorescence parameters data measured in the leaves of Oblačinska sour cherry ecotypes OS, 18, D6, and BOR under control and drought conditions obtained at 0–9 days after stress (DAS). Significant differences between control and drought treatments were labelled by different letters (ANOVA LSD, *p* ≤ 0.05). Values are represented as means ± SD (*n* = 20); Table S4: Principal component analyses of JIP parameters in the leaves of Oblačinska sour cherry ecotypes under control and drought conditions obtained at 0, 4, 6, 7, 8, and 9 days after stress (DAS); Table S5: Composition and concentrations of media components in the micropropagation of sour cherry ecotypes [55,56,57]; Table S6: Recorded data, technical and calculated parameters of JIP-test according to Strasser et al. [10,53].
